# Combined Mueller matrix imaging and artificial intelligence classification framework for Hepatitis B detection

**DOI:** 10.1117/1.JBO.27.7.075002

**Published:** 2022-07-26

**Authors:** Thi-Thu-Hien Pham, Hoang-Phuoc Nguyen, Thanh-Ngan Luu, Ngoc-Bich Le, Van-Toi Vo, Ngoc-Trinh Huynh, Quoc-Hung Phan, Thanh-Hai Le

**Affiliations:** a International University, School of Biomedical Engineering, HCMC, Ho Chi Minh City, Vietnam; b Vietnam National University Ho Chi Minh City, Ho Chi Minh City, Vietnam; c University of Medicine and Pharmacy, Department of Pharmacognosy, HCMC, Ho Chi Minh City, Vietnam; d National United University, Department of Mechanical Engineering, Miaoli, Taiwan; e Ho Chi Minh City University of Technology (HCMUT), Department of Mechatronics, Ho Chi Minh City, Vietnam

**Keywords:** hepatitis B, HBsAg, Mueller matrix imaging, convolutional neural network, polarimetry

## Abstract

**Significance:**

The combination of polarized imaging with artificial intelligence (AI) technology has provided a powerful tool for performing an objective and precise diagnosis in medicine.

**Aim:**

An approach is proposed for the detection of hepatitis B (HB) virus using a combined Mueller matrix imaging technique and deep learning method.

**Approach:**

In the proposed approach, Mueller matrix imaging polarimetry is applied to obtain 4×4 Mueller matrix images of 138 HBsAg-containing (positive) serum samples and 136 HBsAg-free (negative) serum samples. The kernel estimation density results show that, of the 16 Mueller matrix elements, elements $M_{22}$ and $M_{33}$ provide the best discriminatory power between the positive and negative samples.

**Results:**

As a result, $M_{22}$ and $M_{33}$ are taken as the inputs to five different deep learning models: Xception, VGG16, VGG19, ResNet 50, and ResNet150. It is shown that the optimal classification accuracy (94.5%) is obtained using the VGG19 model with element $M_{22}$ as the input.

**Conclusions:**

Overall, the results confirm that the proposed hybrid Mueller matrix imaging and AI framework provides a simple and effective approach for HB virus detection.

## 1 Introduction

Hepatitis B (HB) affects millions of people around the world every year. According to the World Health Organization (WHO), around 2 billion people have been infected with HB virus (HBV) historically, and the annual chronic HBV infection rate and death toll around the world are ∼296 million and 820,000, respectively.^1^ HBV is transmitted from one person to another via the exchange of body fluids and represents a serious health danger to both the individuals involved and the entire local population.^2^ HBV comprises dual-stranded DNA and DNA polymerase enclosed by an exterior layer of HBsAg protein.^3^^–^^5^ The gold standard tests for HBV diagnosis include polymerase chain reaction (PCR) and enzyme-linked immunoassay (ELISA). However, PCR is time-consuming and expensive, whereas ELISA sometimes produces false positives (FPs) and false negatives (FNs).^6^ Consequently, there is an urgent need for cheaper, faster, and more reliable techniques for detecting HBV at early stage.

Mueller matrix polarimetry (MMP) provides a comprehensive and noninvasive approach for the characterization of microstructures and biological tissues.^7^^,^^8^ Many studies have utilized MMP to characterize the polarization properties of pathological tissues, such as colon cancer,^9^ cervical cancer,^10^ skin cancer,^11^ and liver fibrosis.^12^ Ghosh et al.^13^ proposed a method based on Mueller matrix decomposition for separating the linear birefringence (LB), circular birefringence (CB), linear dichroism (LD), and depolarization (Dep) properties of complex turbid media. Ossikovski^14^ utilized a differential Mueller matrix formalism to extract the optical properties of Dep anisotropic media. In general, the results obtained from these studies confirm that MMP provides a promising approach for a wide range of biosensing and clinical diagnosis applications. Lee et al.^15^ showed that Mueller matrix imaging polarimetry (MMIP) is an effective technique for performing the rapid and precise scoring of collagen in pregnancy to evaluate the preterm birth risk. Liu et al.^16^ used a Mueller matrix imaging ellipsometry (MMIE) technique to perform the rapid, nondestructive, and precise measurement of nanostructure materials. Liu et al.^17^ employed MMIP to observe the phase delay change of mouse oocytes before and after maturation, respectively. Badieyan et al.^18^ showed that MMIP provides a dependable and economic approach for the detection of infectious diseases through identifying and discriminating between different bacterial colonies. Meng et al.^19^ found that the performance of transmission MMIP systems can be significantly improved through the use of spatial filtering. Angelo et al.^20^ utilized MMIP to examine diffuse-scattering phantoms under sinusoidal irradiance of varying spatial frequency. The results showed that the spatial frequency generated diverse effects on the unpolarized intensity, linear polarization, and circular polarization, respectively. Sang et al.^21^ combined MMIP with spatial frequency domain imaging to investigate the effects of polarization on the scattering direction of media with near-surface material anisotropy.

Artificial intelligence (AI) is used in many application domains nowadays, including social media, healthcare, education, finance, autonomous vehicles, and so on. One of the most important datasets in the computer vision field is the ImageNet dataset, which contains around 15 million manually-annotated images distributed over 22,000 different categories.^22^ ImageNet has been used to train and evaluate many convolutional neural network (CNN) models in recent years, including VGG, ResNet, and Xception. It has been shown that these models provide an excellent image classification performance for a wide variety of input images. For example, VGG16 achieved a 92.7% top-5 test accuracy when applied to ImageNet,^23^ whereas ResNet^24^ showed a classification error of just 3.57% and Xception achieved a top-5 accuracy of 94.5%.^25^

The feasibility of combining MMIP with AI technology has attracted significant attention in recent years. Ma et al.^26^ combined MMIP with a hybrid 3D–2D CNN to classify cells and showed that the integration of the two technologies resulted in a significant improvement in the classification performance compared with that achieved using MMIP alone. Li et al.^27^ similarly showed that the combined use of MMIP and a CNN provided an effective means of classifying morphologically-similar algae and cyanobacteria. Liu et al.^28^ classified marine microalgae using a low-resolution MMIP technique and a CNN and showed that the classification accuracy obtained using the whole Mueller matrix image was greater than that achieved using the $M_{11}$ image alone at each resolution level. Ma et al.^29^ combined Muller matrix imaging with the transfer learning technique to achieve the automatic classification of electrospun ultrafine fibers with an accuracy of 96%. Zhao et al.^30^ used a combined MMIP and multiparameter fusion network approach to detect giant cell tumors of bone lesions with an accuracy of 99%.

In a previous study, the present group proposed a polarization technique for characterizing the optical properties of turbid media.^31^^,^^32^ Recently, the same group developed a polarization technique for dengue virus detection^33^ and skin cancer detection using deep learning techniques based on polarization properties.^34^^,^^35^ In this study, a combination of MMIP and AI classification framework was utilized to perform HBV detection in human blood serum samples in the reflectance configuration. The MMIP technique was first employed to extract 4×4 Mueller matrix images of 274 blood serum samples, comprising 138 HBsAg-containing (positive) samples and 136 HBsAg-free (negative) samples, respectively. Then, the differential Mueller matrix formalism was used to extract anisotropic parameters of the serum sample, namely the orientation angle of LB (α), the phase retardation (β), the optical rotation angle (γ), the orientation angle of LD ($\theta_{d}$), the LD (D), the circular dichroism (R), and the Dep index (Δ) and to determine the suitable parameters for distinguishing positive and negative samples. Second, the images of Mueller matrix elements having the greatest discriminatory power between the positive and negative samples (as identified from an inspection of the kernel estimation distribution results) were then taken as the inputs to five different deep learning models, namely Xception, VGG16, VGG19, ResNet 50, and ResNet150. It is noted that the proposed approach in this study based on polarimetry imaging in reflectance configuration provides more versatile information than that based on an absolute value from one single point of the previous studies.^34^^,^^35^ Furthermore, it is more useful for the development of classification algorithms and noninvasive techniques for biosensing applications.

## 2 Differential Mueller Matrix Formalism and Deep Learning Model

### 2.1 Mueller Matrix Formalism

The Mueller matrix of a biological sample has the form^36^

$$M=[\begin{array}{cccc} M_{11} & M_{12} & M_{13} & M_{14}\\ M_{21} & M_{22} & M_{23} & M_{24}\\ M_{31} & M_{32} & M_{33} & M_{34}\\ M_{41} & M_{42} & M_{43} & M_{44} \end{array}]\;=[\begin{array}{cccc} \mathrm{HH}+\mathrm{HV}+\mathrm{VH}+\mathrm{VV} & \mathrm{HH}+\mathrm{HV}-\mathrm{VH}-\mathrm{VV} & \mathrm{PH}+\mathrm{PV}-\mathrm{MH}-\mathrm{MV} & \mathrm{RH}+\mathrm{RV}-\mathrm{LH}-\mathrm{LV}\\ \mathrm{HH}-\mathrm{HV}+\mathrm{VH}-\mathrm{VV} & \mathrm{HH}-\mathrm{HV}-\mathrm{VH}+\mathrm{VV} & \mathrm{PH}-\mathrm{PV}-\mathrm{MH}+\mathrm{MV} & \mathrm{RH}-\mathrm{RV}-\mathrm{LH}+\mathrm{LV}\\ \mathrm{HP}-\mathrm{HM}+\mathrm{VP}-\mathrm{VM} & \mathrm{HP}-\mathrm{HM}-\mathrm{VP}+\mathrm{VM} & \mathrm{PP}-\mathrm{PM}-\mathrm{MP}+\mathrm{MM} & \mathrm{RP}-\mathrm{RM}-\mathrm{LP}+\mathrm{LM}\\ \mathrm{HR}-\mathrm{HL}+\mathrm{VR}-\mathrm{VL} & \mathrm{HR}-\mathrm{HL}-\mathrm{VR}+\mathrm{VL} & \mathrm{PR}-\mathrm{PL}-\mathrm{MR}+\mathrm{ML} & \mathrm{RR}-\mathrm{RL}-\mathrm{LR}+\mathrm{LL} \end{array}],$$ (1)

where H, V, P, M, R, and L denote 0 deg, 90 deg, 45 deg, 135 deg, circular right-hand, and circular left-hand polarization states, respectively, and each two-letter combination indicates the experimental settings required to obtain the corresponding Mueller matrix element. For instance, the state (HV) indicates the use of linear and horizontal polarization light, respectively. Thus, to obtain Mueller matrix element $M_{13}$, e.g., four measurements are required, namely (PH), (PV), (MH), and (MV). A detailed inspection of Eq. (1) reveals that a total of 36 measurements are needed to construct the full Mueller matrix.

The differential Mueller matrix for extracting optical properties of anisotropic samples was developed and described in detail in Ref. 37. This method is a further extension of the conventional differential Mueller matrix introduced first by Azzam.^38^ Briefly, the differential Mueller matrix of a biological sample with light propagating along the z-axis of a right-handed Cartesian coordinate system is written as^38^

$$m=(\mathrm{dM}/\mathrm{dz})M^{-1}=V_{M}(\frac{\ln(\lambda_{M})}{z})V_{M}^{-1}=[\begin{array}{cccc} m_{11} & m_{12} & m_{13} & m_{14}\\ m_{21} & m_{22} & m_{23} & m_{24}\\ m_{31} & m_{32} & m_{33} & m_{34}\\ m_{41} & m_{42} & m_{43} & m_{44} \end{array}],$$ (2)

where $V_{M},\lambda_{M}$ are the eigenvector and eigenvalue, respectively, of the Mueller matrix, M. As discussed in Ref. 37, the anisotropic parameters of a biological sample include the orientation angle of LB α, the phase retardation β, the optical rotation angle γ, the orientation angle of LD $\theta_{d}$, the LD D, the circular dichroism R, and the Dep index Δ. These parameters are expressed in terms of the elements of the differential Mueller matrix as follows:

$$\alpha =\frac{1}{2}\;\tan^{-1}(\frac{m_{42}-m_{24}}{m_{34}-m_{43}}),$$ (3)

$$\beta =\sqrt{{\left[\frac{\left(m_{42}-m_{24}\right)}{2}\right]}^{2}+{\left[\frac{\left(m_{34}-m_{43}\right)}{2}\right]}^{2}},$$ (4)

$$\gamma =\frac{\left(m_{23}-m_{32}\right)}{4},$$ (5)

$$\theta_{d}=\frac{1}{2}\;\tan^{-1}(\frac{m_{13}+m_{31}}{m_{12}+m_{21}}),$$ (6)

$$D=\frac{1-e^{-2\sqrt{{\left(m_{12}+m_{21}\right)}^{2}+{\left(m_{13}+m_{31}\right)}^{2}}}}{1+e^{-2\sqrt{{\left(m_{12}+m_{21}\right)}^{2}+{\left(m_{13}+m_{31}\right)}^{2}}}},$$ (7)

$$R=\frac{e^{\left(\frac{m_{14}+m_{41}}{2}\right)}-1}{e^{\left(\frac{m_{14}+m_{41}}{2}\right)}+1},$$ (8)

$$\Delta =1-\sqrt{\frac{K_{22}^{2}+K_{33}^{2}+K_{44}^{2}}{3}},\;0\leq \Delta \leq 1,$$ (9)

where $K_{22}=m_{22}-m_{11}$ and $K_{33}=m_{33}-m_{11}$ are the degrees of linear Dep, and $K_{44}=m_{44}-m_{11}$ is the degree of circular Dep. Then, the seven anisotropic parameters that are extracted from Eqs. (3)–(9) are used as the comparison in terms of their discriminatory powers between positive and negative HBV samples.

### 2.2 Deep Learning Model

**Fig. 1:**
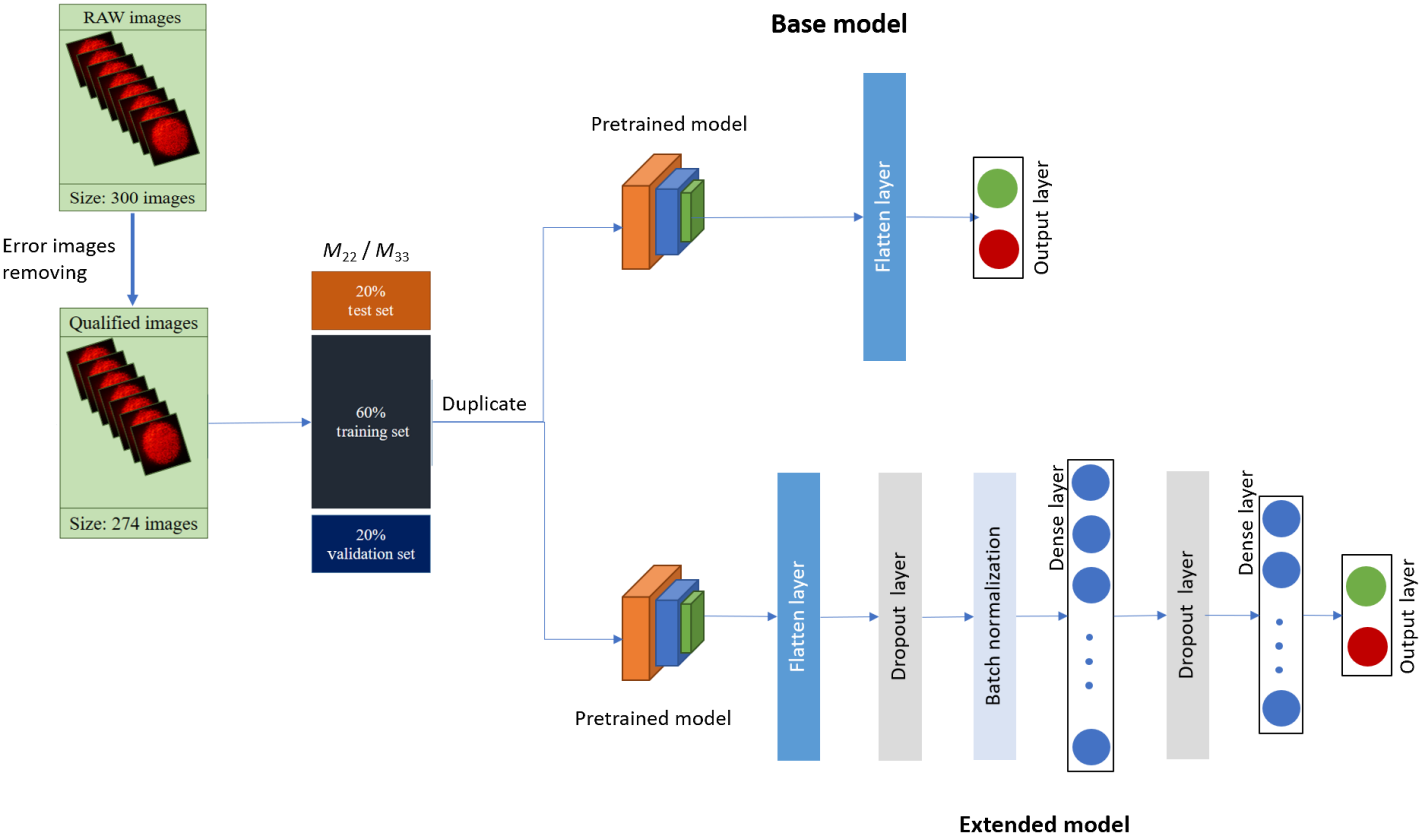
Schematic of deep learning model architecture.

In the present study, the positive and negative HBV samples were classified using five deep learning models based on the MMIP-derived Mueller matrix elements (see Sec. 4.2). Figure 1 shows the basic architecture of the deep learning models implemented in the present study. (Note that the models were all implemented on Google Colab Pro with a Tesla P100 GPU.)

For each model, 274 samples were taken as the input to the learning algorithm, with 219 samples used for training and validation purposes (i.e., 80% of the dataset) and 55 images retained for testing (i.e., 20% of the dataset). It is noted that, for the training set of MMIP images, a fivefold cross-validation technique was applied, and for solving the problem of insufficient training data, a transfer learning technique was applied in this study.^39^ Furthermore, the augmentation technique was applied to increase the diversification of the dataset during the training process. As shown in Fig. 1, two model variants were considered in each case: a base model and an extended model. In the base model, all of the layers were frozen, i.e., the weights pretrained on ImageNet were not modified but were used to classify the input MMIM images directly. By contrast, in the extended model, the layers were unfrozen and were thus updated during the training process in accordance with the loss function. It is noted that the dense layers were added to slowly reduce the output of the last layer of models from 1000 classes to [256, 128, 64, 32, 16] (i.e., intermediate layers) and finally to two classes. Moreover, dropout and batch normalization layers were put together with fully-connected layers (i.e., dense layers) in the model architecture to reduce overfitting. For both model variants, the binary cross entropy loss was employed, with an initial learning rate of 0.0001, the Adam optimizer, and a batch size of 32. Moreover, the classification performance was evaluated using four metrics, namely,

$$\text{Accuracy}=\frac{\mathrm{TP}+\mathrm{TN}}{\mathrm{TP}+\mathrm{TN}+\mathrm{FP}+\mathrm{FN}},$$ (10)

$$\text{Precision}=\frac{\mathrm{TP}}{\mathrm{TP}+\mathrm{FP}},$$ (11)

$$\text{Recall}=\frac{\mathrm{TP}}{\mathrm{TP}+\mathrm{FN}},$$ (12)

$$F1\text{ score}=2\times \frac{\text{Precision}\times \text{Recall}}{\text{Precision}+\text{Recall}},$$ (13)

where TP, TN, FP, and FN denote true positive, true negative, false positive, and false negative, respectively.

Machine learning algorithms are highly susceptible to the range and distribution of the attribute values. In particular, data outliers can harm and delude the training process, resulting in prolonged training intervals and, ultimately, a poorer result. Thus, detecting and removing outliers in the input data is of crucial importance in improving the classification performance of the algorithm.^40^ One of the most commonly used methods for identifying outliers is the Tukey test,^41^ in which the outliers are defined based on the quartiles of the data, where the first quartile $Q_{1}$ is the value larger than a quarter of the data, the second quartile $Q_{2}$ (the median) is the value larger than half of the data, and the third quartile $Q_{3}$ is the value larger than three-quarters of the data. The interquartile range is defined as $\mathrm{IQR}=Q_{3}-Q_{1}$, and the outliers are then defined in accordance with Tukey’s rule as

$$\left\{ \begin{array}{l} \text{Outliers}<Q_{1}-1.5\times \mathrm{IQR}\\ Q_{3}+1.5\times \mathrm{IQR}<\text{Outliers} \end{array},\right.$$ (14)

where IQR stands for the interquartile range ($Q_{3}-Q_{1}$).

## 3 Sample Preparation and Experimental Setup

### 3.1 Sample Preparation

**Fig. 2:**
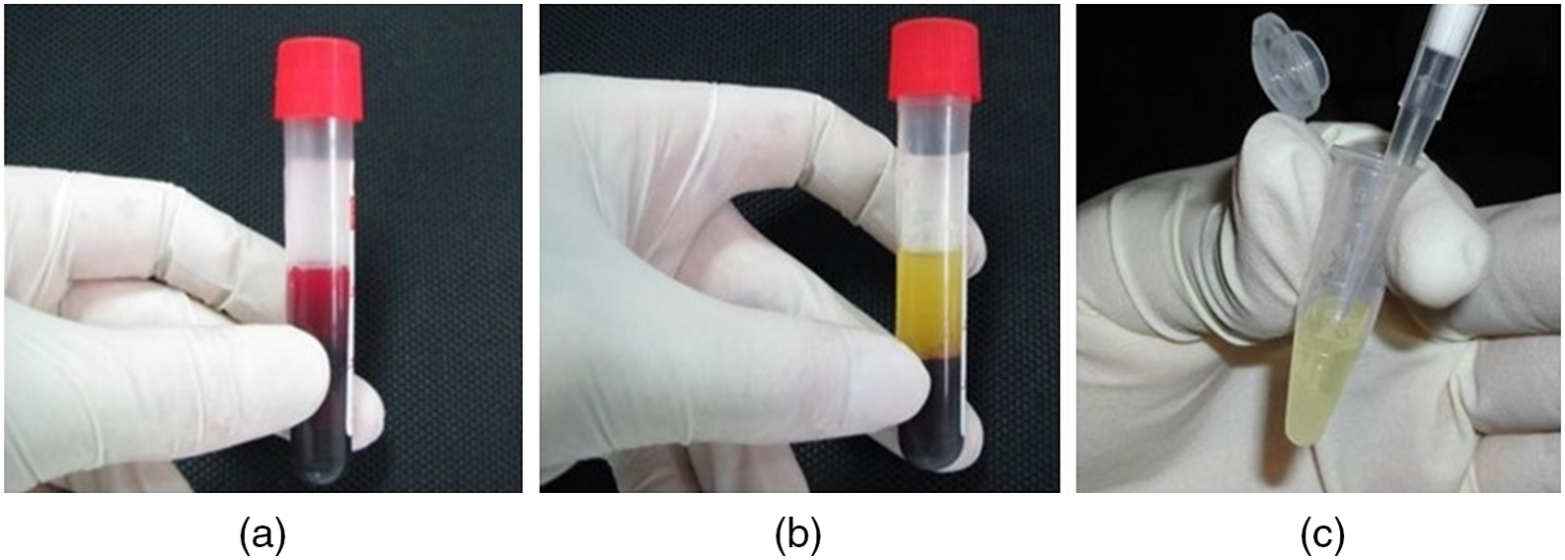
Blood sample: (a) before and (b) after centrifuging. (c) Final hepatitis serum.

A total of 274 human serum samples were obtained from the General Central Hospital of Tien Giang Province in Vietnam between May and June 2020 [see Fig. 2(a)]. According to clinical assays, 138 of the samples were positive for HBsAg (an antigen for HBV), and 136 samples were negative. The samples were placed in serum separator tubes spray-coated with silica to assist in clotting and a polymer gel for separating the serum. The tubes were stored vertically for 20 to 30 min to form blood clots and were then centrifuged at 4000 to 5000 rpm for 10 min to separate the serum layer [see Fig. 2(b)]. The serum was extracted by a sterile plastic pipette and placed in 1.5 mL Eppendorf tubes [see Fig. 2(c)]. Finally, the tubes were stored in 100-position cryo-boxes at −20°C until required for use. The entire process was performed under the approval of the Ethics Institute of the hospital involved.

Prior to the MMIP tests, two cuvettes were prepared: one for the positive samples and one for the negative samples. The cuvettes were soaked in medical alcohol at a temperature of 70°C for 15 min, rinsed with distilled water, and then left to dry. A clean micropipette was used to transfer the sample (positive or negative) from the Eppendorf tube to the cuvette. Finally, the cuvette was sealed and placed in the holder of the MMIP measurement system to evaluate its Dep properties.

### 3.2 Experimental Setup

**Fig. 3:**
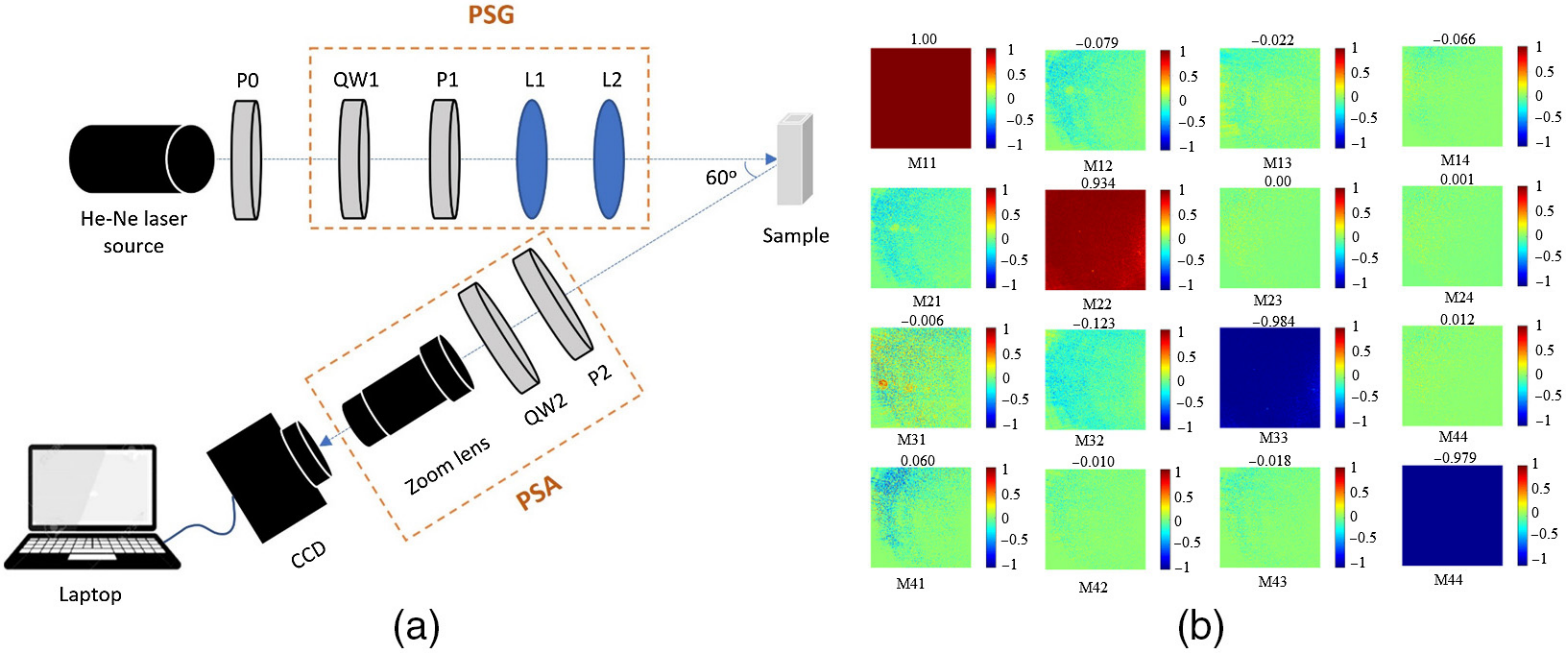
(a) Schematic of experimental MMIM setup and (b) calibration result of measured Mueller matrix of a standard mirror.

Figure 3 presents a schematic illustration of the experimental setup. As shown, the system consists mainly of a He–Ne laser as the light source (Thorlabs Inc. HRS015B, 633 nm), a polarizer (P0, Thorlabs Inc. LPVIS100-MP), a polarization state generator (PSG), a polarization state analyzer (PSA), a zoom lens (Thorlabs Inc. MVL6X12Z), a charge-coupled device (CCD) camera, and a computer. It is also noted that a coherent light source was used for the sake of simplicity and stability. The PSG creates polarized light from the unfiltered laser source, while the PSA analyzes the polarization state of the light beam scattered from the sample. The PSG comprises a quarter waveplate (QW1, Thorlabs Inc. WPQ05M-633) to generate circular polarization light, a linear polarizer (P1, Thorlabs Inc. LPVIS100-MP) to produce linear polarization light, and two condenser lenses (L1, L2, Thorlabs Inc. LSSB04-A) to focus the light onto the sample. Meanwhile, the PSA consists of a quarter waveplate (QW2, Thorlabs Inc. WPQ05M-633) and a linear polarizer (P2, Thorlabs Inc. LPVIS100-MP). In performing the measurement process, the incident angle was set to 60 deg to prevent the reflection of the incident light from the sample surface and to obtain a good polarization image.^33^^,^^42^ Moreover, the polarizers (P1, P2) and quarter waveplates (QW1, QW2) in the PSG and PSA were mounted on rotators (Sigma Koki Co., SGSP-60YAW-0B) to generate the 36 polarization states required to construct the Mueller matrix of each sample. In the PSG, the linear polarization states (0 deg, 45 deg, 90 deg, and 135 deg) were generated simply by rotating the polarizer (P1). The circular polarization lights (right and left) were produced by moving P1 out of the laser path with a slider and rotating the QW1 to the right- and left-hand circular polarization states. Similarly, in the PSA, the linear states of polarization were produced by rotating polarizer P2 and moving QW2 out of the laser path with a slider, whereas the circular polarization lights were generated by rotating QW2 and moving P2 out of the laser path with a slider. The principal axis angle of optical elements in the measurement system and the degree of Dep were calibrated and controlled by a commercial Stokes polarimeter (Thorlabs Inc., PAX5710). A similar calibration process was described in detail in Refs. 31 and 32. The degree of polarization of the output light is measured by commercial Stokes polarimeter and is approximately 99.99%. The calibration result of the measured Mueller matrix of a standard mirror (Thorlabs Inc., BB1-E02) with an accuracy of $10^{-2}$ is shown in Fig. 3(b). It is noted that the measurement system was first developed by the Hui Ma group^7^^,^^8^^,^^43^ for characterizing the microstructure of biological tissue. Furthermore, the system was also employed by the present group for dengue detection.^33^ Thus, the feasibility of the measurement for extracting the Mueller matrix of anisotropic turbid media is confirmed. When performing the experiments, HBV samples were stored in a 1.3 mm-thickness quartz cuvette (Thorlabs Inc., CV10Q35F). It is noted that both the incident photon beam and the remission photon beam went through the isotropic cuvette sample holder. Subsequently, blood plasma is an anisotropic scattering medium but is contained in an isotropic cylinder. Therefore, the Muller matrix strongly depends on the angle at which the detector is set relative to the cuvette. The phenomenon of using an isotropic cuvette for anisotropic samples is common and well known. The simple way to eliminate the effect of the cuvette material is by dividing the measured results by the results obtained by the cuvette itself. In the current setup, the Mueller matrix is measured with 36 images. It is noted that the Mueller matrix is able to be constructed with 16 images but requires a more complicated system.^44^

## 4 Results and Discussion

### 4.1 Anisotropic Properties of Serum Samples

**Fig. 4:**
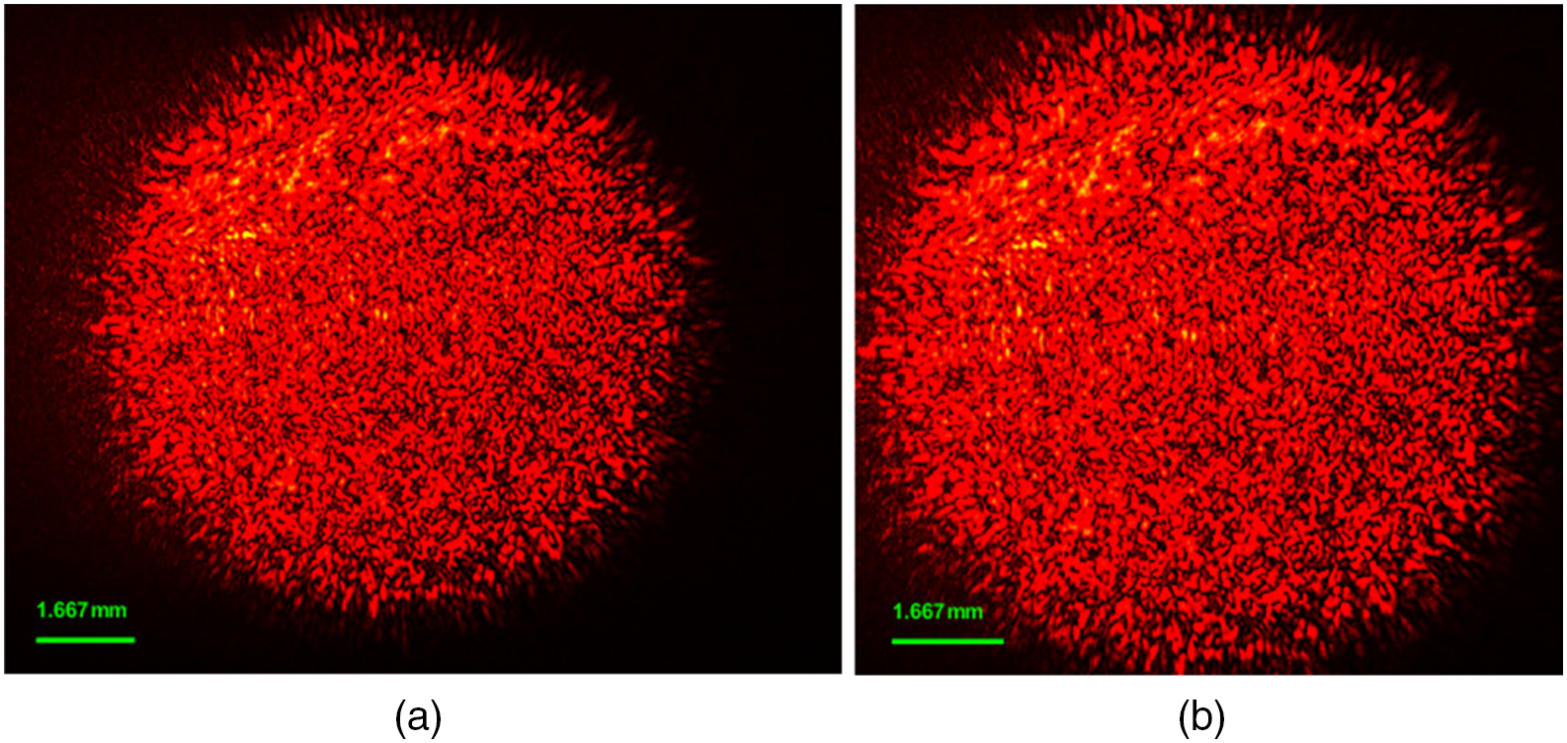
HBV images (a) before and (b) after dropping.

Figure 4 shows the results of HBV images before and after dropping, respectively. The original image captured from a CCD camera has the size of 1280×1024  pixels. For the dropping step, an average kernel was created as large as the sample (800×800). It is noted that the size of the kernel was chosen after numerous trial and error efforts. The kernel swept across every pixel of each image. After that, the largest average intensity value was chosen, which is normally the center pixel of the image. From the center pixel, the image spread to the size of 900×900 (i.e., 450 pixels in each direction). As a result, a “for” loop was used to automatically crop 274 samples (with 36 images for each sample) and save new images in PNG format.

**Fig. 5:**
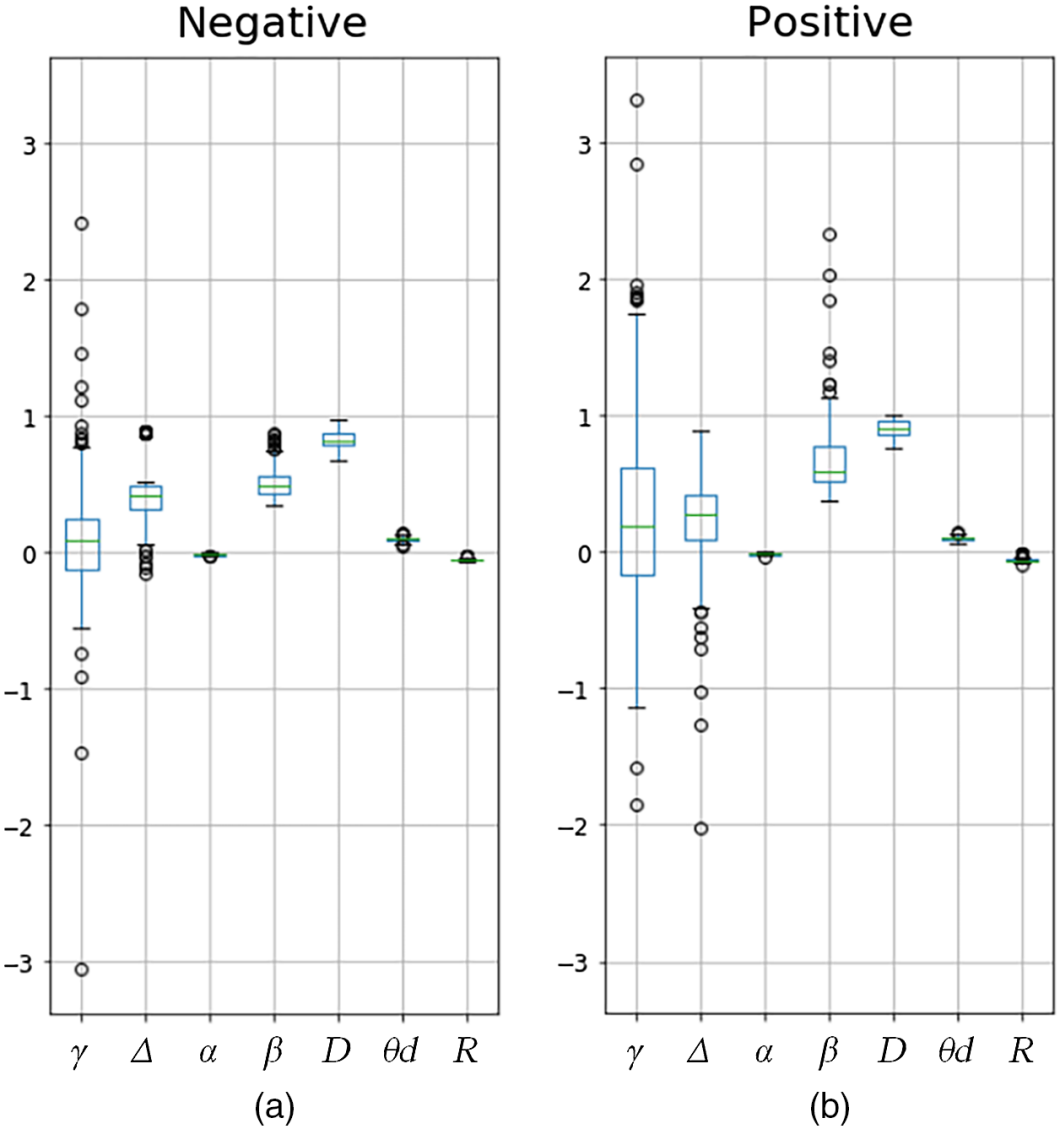
Seaborn boxplot of anisotropic parameters of serum samples: (a) negative sample and (b) positive sample.

Table 1 and Fig. 5 show the values and seaborn boxplots of the anisotropic parameters of the negative and positive samples. As shown, the values of Δ provide a good discriminatory power between two samples because of the scattering properties of blood plasma. The values of Δ have a value overlap only in the range of 0.32 to 0.42, and the outliers of the positive class are much lower than those of the negative class. The value β also provides a reliable indication of the sample class because of the photoelasticity properties of possible fiber structure within blood plasma. The ranges of the two classes have a minor overlap (between 0.51 and 0.55), and the outliers of the positive class have a higher value than those of the negative class. Parameters D and R can also be used to discriminate between the samples possibly containing the protein structure of antibodies (IgG or IgM) within the samples generating the dichroism properties. The values of D and R have overlaps between the two classes (i.e., from 0.86 to 0.876 and −0.059 to −0.054, respectively). In contrast, the value range of γ, α, and $\theta_{d}$ cannot be used to reliably distinguish between the two samples. γ is a well-known parameter used for diabetes measurement, and α and $\theta_{d}$ are parameters for collagen and tumor structure, respectively. As shown, the outlier values of γ also fall within a similar range for both samples. The value ranges of α and $\theta_{d}$ are almost the same for both classes.

**Table 1:** Anisotropic parameters of negative and positive serum samples.

Sample		Parameters						
		γ	Δ	α	β	θ	D	R
Negative	Mean	0.106	0.440	−0.015	0.509	0.099	0.836	−0.053
	Std	0.525	0.240	0.005	0.110	0.015	0.061	0.009
	Min	−3.054	−0.156	−0.030	0.350	0.048	0.679	−0.073
	Max	2.412	0.892	−0.002	0.870	0.150	0.977	−0.021
	$Q_{1}$	−0.118	0.319	−0.018	0.428	0.091	0.790	−0.060
	$Q_{3}$	0.243	0.492	−0.011	0.558	0.109	0.877	−0.048
	IQR	0.361	0.173	0.007	0.130	0.018	0.086	0.011
Positive	Mean	0.317	0.230	−0.013	0.700	0.103	0.905	−0.056
	Std	0.765	0.431	0.006	0.300	0.014	0.060	0.014
	Min	−1.856	−2.016	−0.032	0.372	0.069	0.763	−0.098
	Max	3.318	0.892	−0.0001	2.335	0.143	1.000	−0.011
	$Q_{1}$	−0.161	0.091	−0.017	0.513	0.093	0.860	−0.065
	$Q_{3}$	0.623	0.423	−0.009	0.776	0.112	0.958	−0.054
	IQR	0.784	0.332	0.008	0.263	0.019	0.098	0.011

### 4.2 Application of Deep Learning Models to HBV Detection

**Fig. 6:**
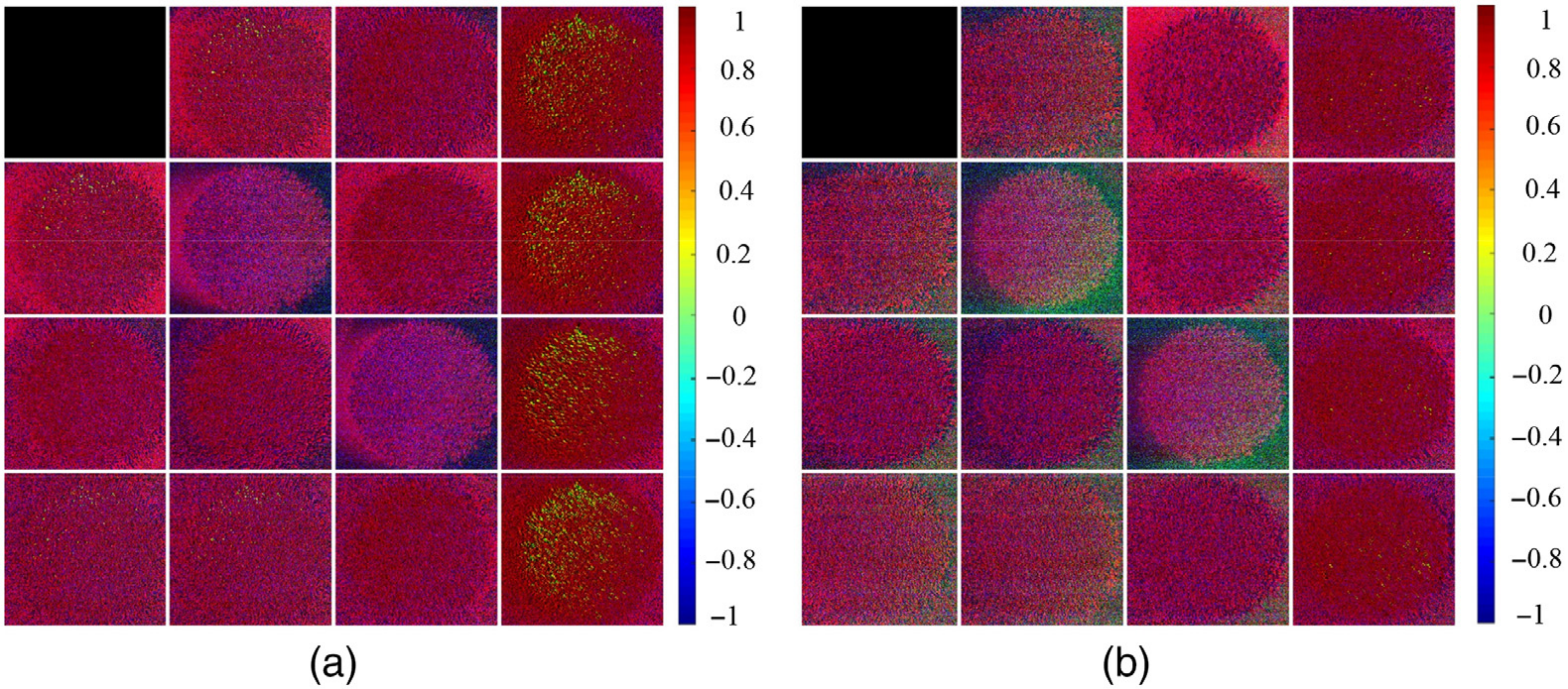
4×4 Mueller matrix images in BGR color format: (a) negative sample and (b) positive sample.

**Fig. 7:**
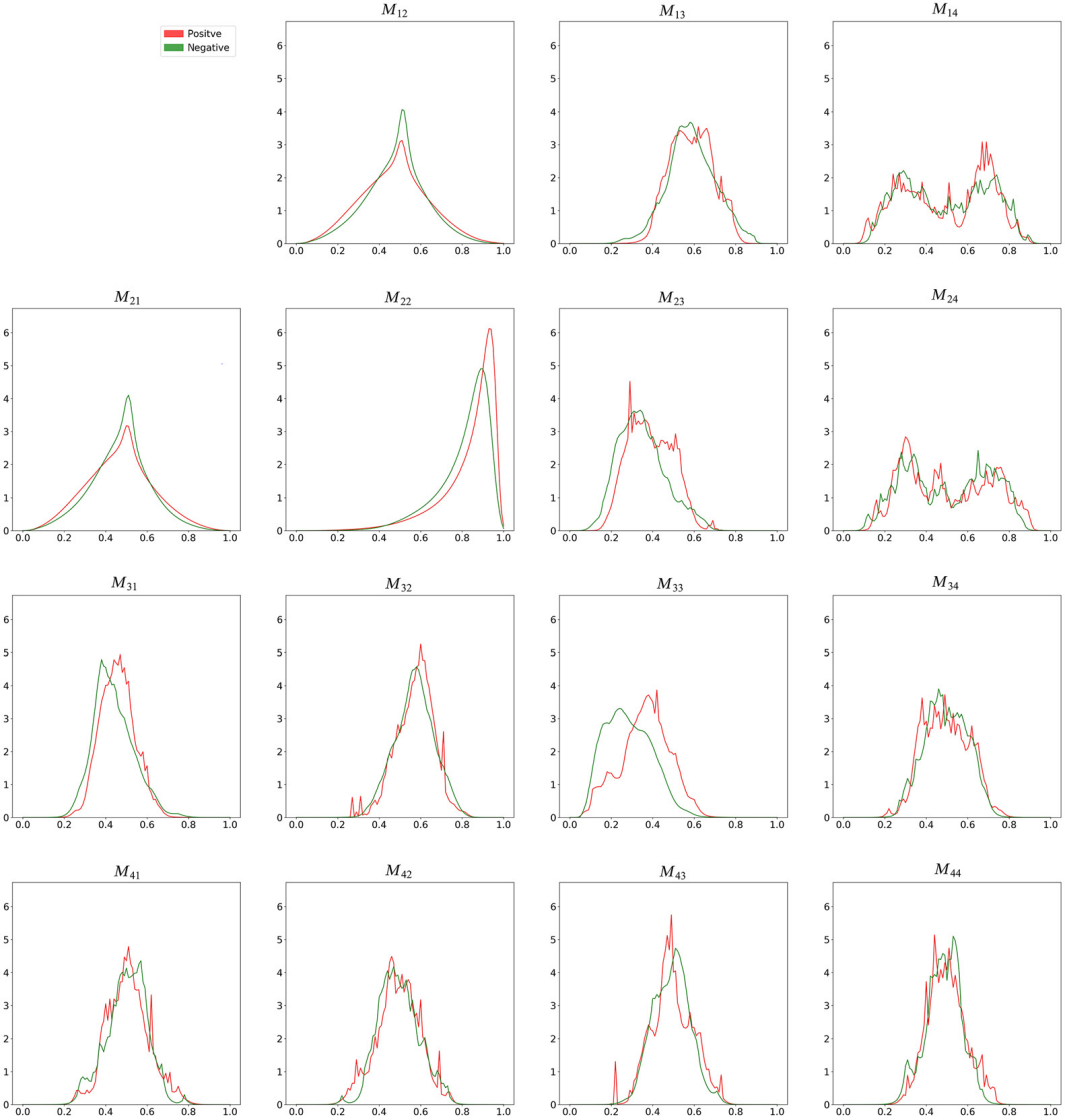
Kernel density estimations of positive and negative samples.

Figures 6(a) and 6(b) present illustrative 4×4 Mueller matrix images of the negative and positive HbsAg samples, respectively. Figure 7 presents the corresponding kernel density estimation results. The images presented in Fig. 6 confirm that qualitative differences exist between the Mueller matrix element images of the two classes. A close inspection of Fig. 7 reveals that elements $M_{22}$ and $M_{33}$ show the greatest difference between the two classes and hence provide the most reliable elements for differentiating between them. It is noted that these results show a good quantitative agreement with those obtained from Ref. 33 and are consistent with the results reported in Ref. 45. Accordingly, two datasets consisting of $M_{22}$ and $M_{33}$ images, respectively, were prepared and supplied as inputs to five different deep learning models (Xception, VGG16, VGG19, ResNet50, and ResNet150).

Table 2 shows the number of positive and negative HbsAg samples used for training and testing. From 138 of the positive and 136 negative samples, 219 samples (i.e., 108 positive samples and 111 negative samples) were used for training with a fivefold cross-validation technique, and 55 samples (i.e., 30 positive samples and 25 negative samples) were used for testing.

**Table 2:** Number of positive and negative HbsAg samples in training and testing datasets.

	Positive	Negative
Number of training samples	108	111
Number of testing samples	30	25

### 4.3 Base Model Results

**Fig. 8:**
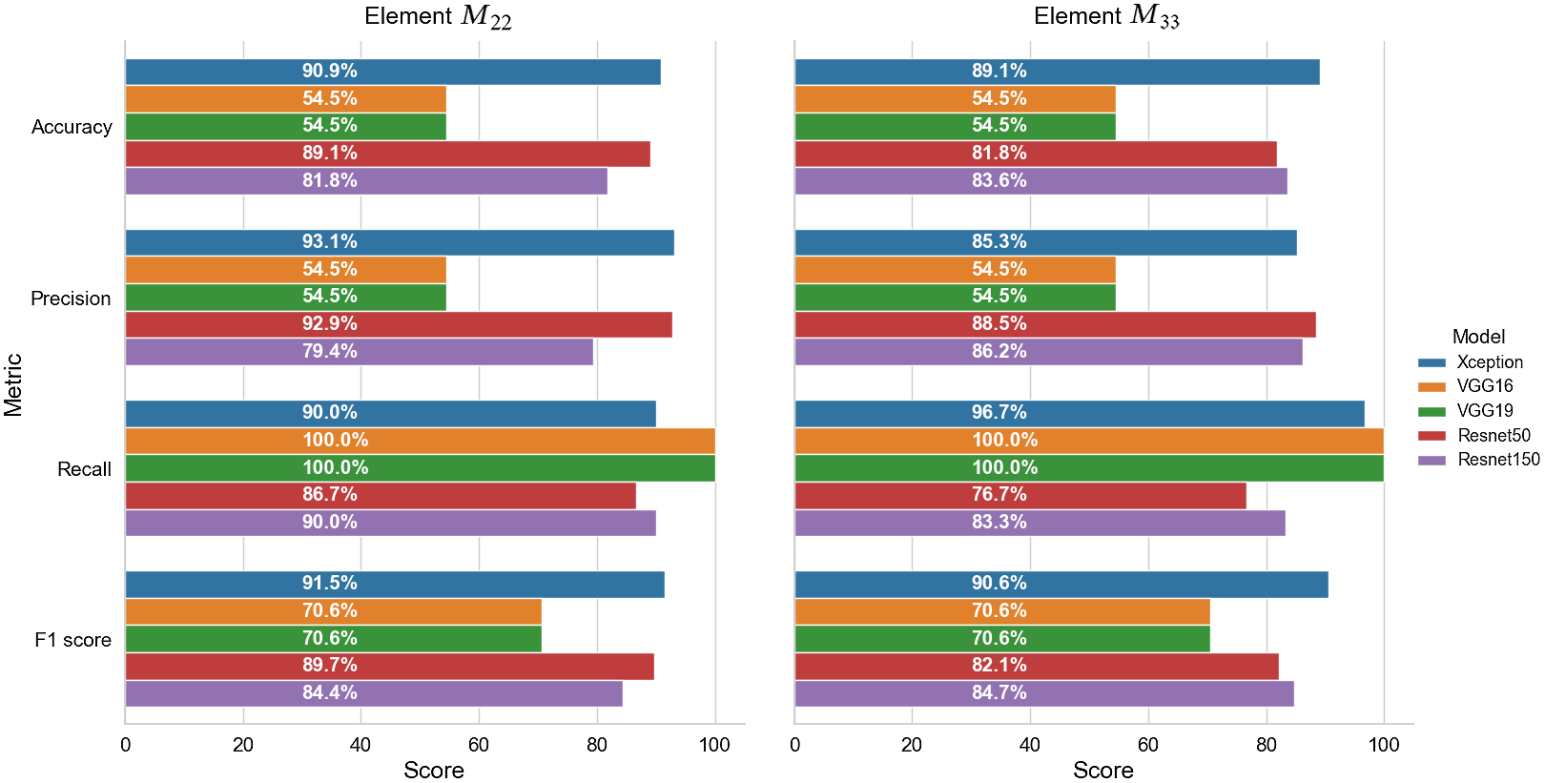
Performance metrics of five base models when applied to the testing dataset.

Figure 8 shows the performance metrics of the five base models when applied to the test dataset using matrix elements (a) $M_{22}$ and (b) $M_{33}$ as the input for classification purposes. Obviously, as shown in Fig. 8, the abilities of detection among the five models have significant differences. It is seen that the Xception, ResNet50, and ResNet150 models all have accuracies of >80%. By contrast, the two VGG models have an accuracy of just 54.5%. Moreover, both models have a recall score of 100%, which indicates that they consider all of the healthy samples to be HBV samples.

**Fig. 9:**
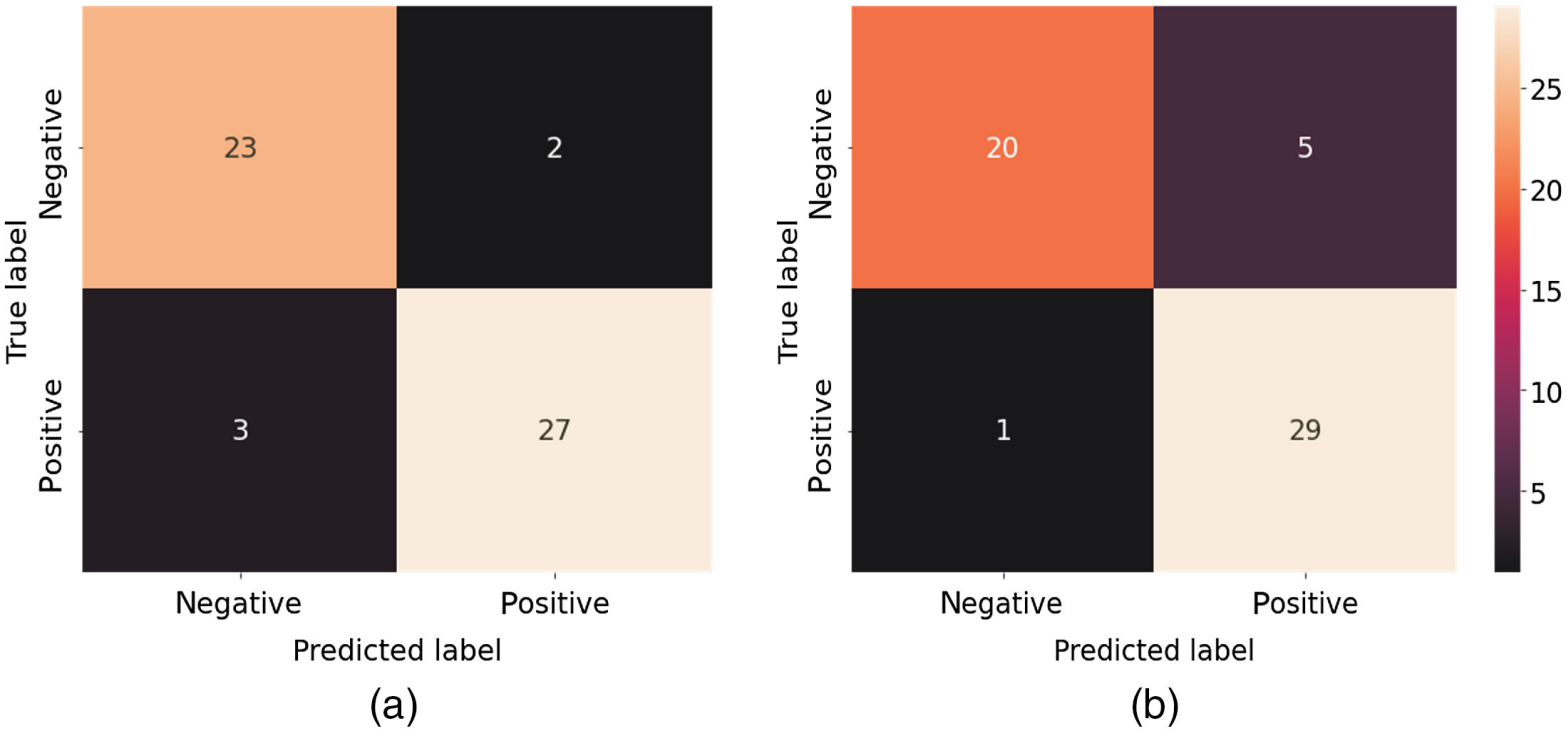
Confusion matrixes for base the Xception model using (a) $M_{22}$ and (b) $M_{33}$ as inputs.

Of all models, the Xception model provides the most stable performance across the five performance metrics and achieves the highest accuracy of 90.9% and 87.3% for matrix elements $M_{22}$ and $M_{33}$, respectively. Referring to the confusion matrixes in Fig. 9, it is seen that matrix element $M_{22}$ results in five incorrect detection cases (i.e., three FN and two FP), whereas matrix element $M_{33}$ results in six incorrect detection cases (i.e., one FN and five FP). However, matrix element $M_{33}$ results in only one positive sample being incorrectly classified as a negative (i.e., normal) sample. It is noted that, in a medical procedure of diagnosis, a highly sensitive test is when there are few FN results; in other words, few actual cases are missed.^46^ Therefore, usually, the prediction model with a low false negative rate will be selected.

**Fig. 10:**
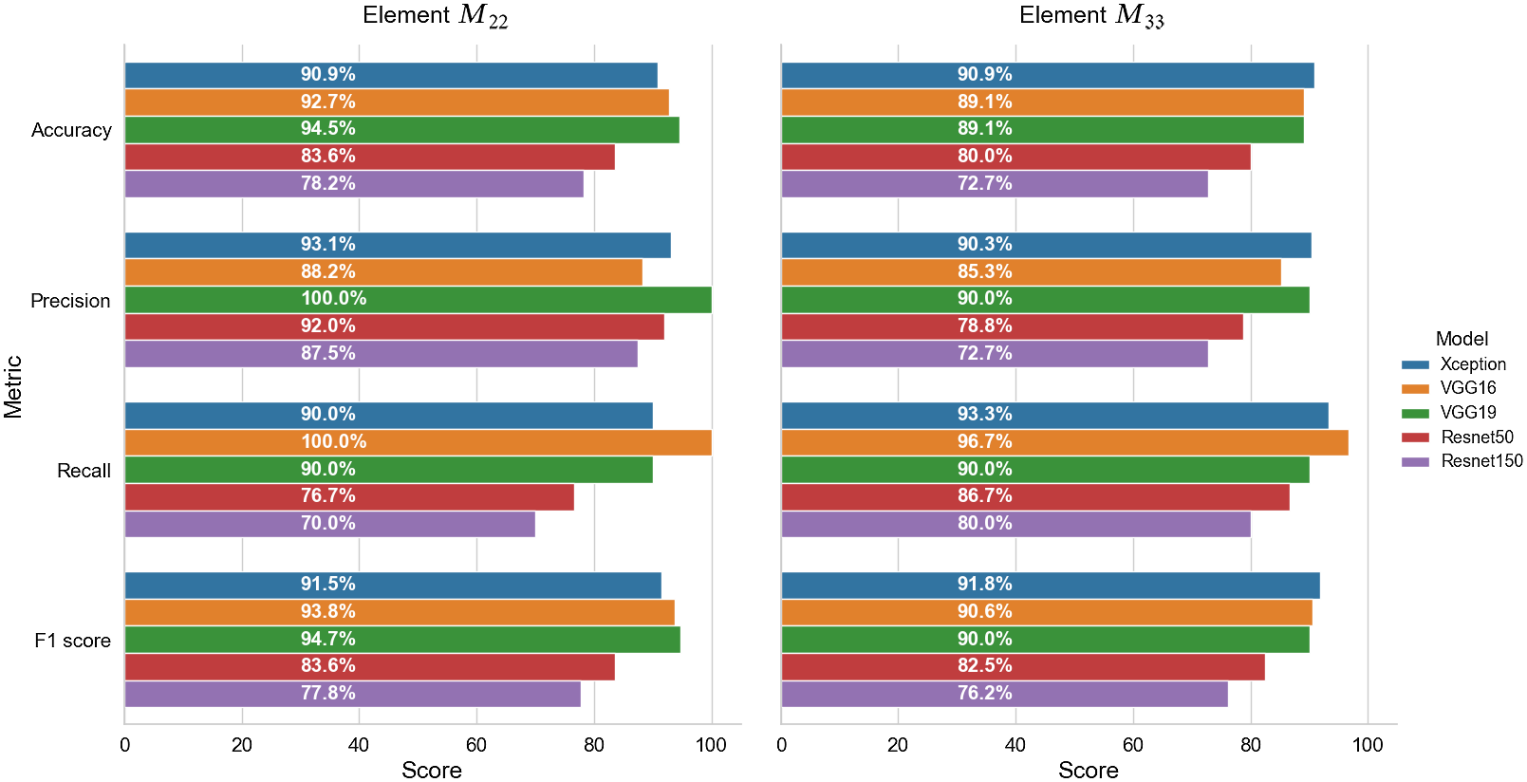
Performance metrics of the five extended models when applied to the testing dataset.

As described in Sec. 2, the base models were extended through the addition of a dropout layer, a batch normalization layer, and fully-connected layers. Keras callbacks (ModelCheckpoint, EarlyStopping, and GridsearchCV) were additionally used to optimize the training procedure. These callbacks are used to test different fully connected layer configurations with output features of [256, 128, 64, 32, 16], $L_{2}$ regularization, and kernel constraint automatically. Figure 10 shows the performance metrics of the extended models with the best output features of 32 for a fully connected layer when using matrix elements (a) $M_{22}$ and (b) $M_{33}$ as the basis for the classification process.

The Xception, VGG16, and VGG19 models all achieve an F1 score of >90% for both matrix elements. For the case in which $M_{22}$ is taken as the basis for the classification process, the VGG19 model achieves the highest accuracy (94.5%) and F1 score (94.7%), whereas the Xception model yields the lowest accuracy (90.9%) and F1 score (91.5%). By contrast, when using element $M_{33}$ as the input, the Xception model achieves the highest F1 score (91.8%), whereas the VGG19 model achieves the lowest score (90.0%).

The ResNet models achieve a lower classification performance than the VGG and Xception models. However, the ResNet150 and ResNet50 models nevertheless achieve precision scores of 87.5% and 92%, respectively, when taking matrix element $M_{22}$ as the input to the classification process. It is noted that, in this study when using elements $M_{22}$ and $M_{33}$ as the inputs, the performance of base ResNet models achieves better results than the extended ones. This can be explained by the addition of some layers to reduce output features slowly did not guarantee an improvement in the performance of the pretrained models.

**Fig. 11:**
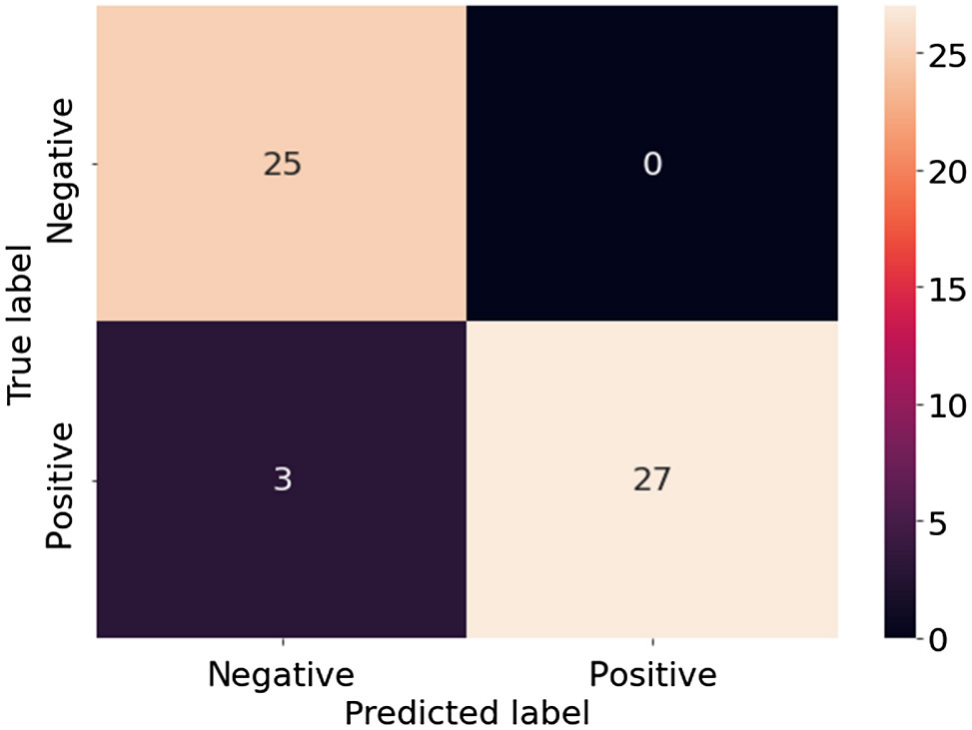
Confusion matrix of extended the VGG19 model using $M_{22}$ as input.

Figure 11 shows the confusion matrix of the extended VGG19 model when using matrix element $M_{22}$ as the input. As shown, all 25 negative samples are correctly classified, giving a precision score of 100% (see Fig. 10). However, 3 of the 30 positive samples are not recognized, leading to a recall score of 90%.

**Fig. 12:**
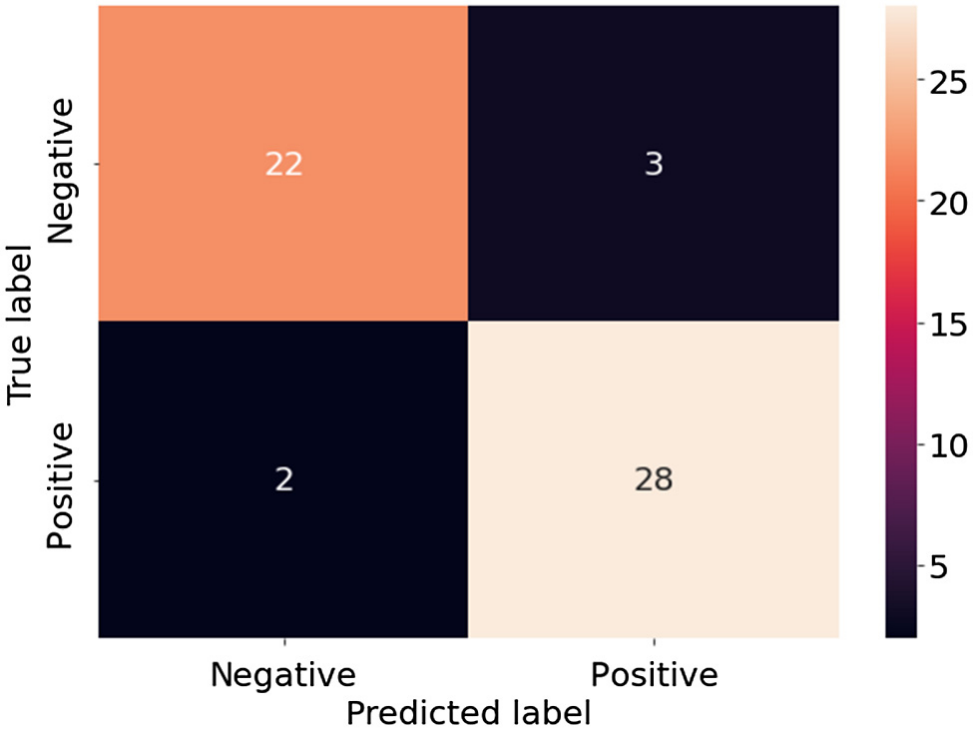
Confusion matrix of extended the Xception model using $M_{33}$ as input.

Figure 12 shows the confusion matrix of the extended Xception model when using matrix element $M_{33}$ as the input. It is seen that just three negative samples and two positive samples are misclassified. Thus, the precision and recall scores are equal to 90.3% and 93.3%, respectively, and the overall accuracy is 90.9%.

## 5 Conclusion

This study has proposed a combined MMIM and machine learning framework for performing the detection of HBV based on the polarization properties of blood serum samples. The results have shown that, among all of the optical anisotropic parameters of HBV serum samples, parameters Δ, β, D, and R provide the optimal discriminatory power between the negative and positive classes. Furthermore, five deep learning models have been considered: Xception, VGG16, VGG19, ResNet 50, and ResNet150. For each model, two variants have been implemented, namely a base model with fixed weights based on a pretrained ImageNet model and an extended model in which the weights are adjusted adaptively over the course of the training process. The results have shown that elements $M_{22}$ and $M_{33}$ of the Mueller matrix provide the maximum discriminatory power between the negative and positive samples. Moreover, among the five base models, the Xception model achieved the highest accuracy of 90.9% and 87.3% when using matrix elements $M_{22}$ and $M_{33}$ for classification purposes, respectively. By contrast, for the extended models, the optimal accuracy (94.5%) was obtained using the VGG19 model with element $M_{22}$ as the input. Overall, the results indicate that the framework proposed in this study provides a reliable and straightforward approach for detecting HBV.

## Code, Data, and Materials Availability

No materials were used for the analysis. The code and data used to generate the results are available in the Code Ocean repository: https://codeocean.com/capsule/9493796/tree.
